# Genetic Diversity and Expanded Phenotypes in Dystonia: Insights From Large‐Scale Exome Sequencing

**DOI:** 10.1002/acn3.70100

**Published:** 2025-06-18

**Authors:** Mirja Thomsen, Fabian Ott, Sebastian Loens, Gamze Kilic‐Berkmen, Ai Huey Tan, Shen‐Yang Lim, Ebba Lohmann, Kaja M. Schröder, Lea Ipsen, Lena A. Nothacker, Linn Welzel, Alexandra S. Rudnik, Frauke Hinrichs, Thorsten Odorfer, Kirsten E. Zeuner, Friederike Schumann, Andrea A. Kühn, Simone Zittel, Marius Moeller, Robert Pfister, Christoph Kamm, Anthony E. Lang, Yi Wen Tay, Ana Luísa de Almeida Marcelino, Marie Vidailhet, Emmanuel Roze, Joel S. Perlmutter, Jeanne S. Feuerstein, Victor S. C. Fung, Florence Chang, Richard L. Barbano, Steven Bellows, Aparna A. Wagle Shukla, Alberto J. Espay, Mark S. LeDoux, Brian D. Berman, Stephen Reich, Andres Deik, Andre Franke, Michael Wittig, Sören Franzenburg, Jens Volkmann, Norbert Brüggemann, H. A. Jinnah, Tobias Bäumer, Christine Klein, Hauke Busch, Katja Lohmann

**Affiliations:** ^1^ Institute of Neurogenetics University of Lübeck Lübeck Germany; ^2^ Medical Systems Biology Division, Institute of Experimental Dermatology University of Lübeck Lübeck Germany; ^3^ Institute of Systems Motor Science University of Lübeck Lübeck Germany; ^4^ Department of Neurology Emory University School of Medicine Atlanta Georgia USA; ^5^ Division of Neurology, Department of Medicine Faculty of Medicine, University of Malaya Kuala Lumpur Malaysia; ^6^ Hertie Institute for Clinical Brain Research, University of Tübingen Tübingen Germany; ^7^ German Center for Neurodegenerative Diseases (DZNE) Tübingen Germany; ^8^ Department of Neurology University Hospital Würzburg Würzburg Germany; ^9^ Department of Neurology University Hospital Schleswig‐Holstein, Campus Kiel Kiel Germany; ^10^ Department of Neurology Charité—Universitätsmedizin Berlin Berlin Germany; ^11^ Department of Neurology University Medical Center Hamburg‐Eppendorf Hamburg Germany; ^12^ Neurological Practice Neusäß Germany; ^13^ Department of Neurology University Medical Center Rostock Rostock Germany; ^14^ Edmond J. Safra Program in Parkinson's Disease, the Rossy PSP Centre and Department of Medicine (Neurology), Toronto Western Hospital University of Toronto Toronto Ontario Canada; ^15^ Department of Biomedical Science Faculty of Medicine, University of Malaya Kuala Lumpur Malaysia; ^16^ Sorbonne University, Paris Brain Institute (ICM), Inserm, CNRS, and Center of Excellence of Neurodegenerative Disease (CoEN), AP‐HP, Pitié‐Salpêtrière Hospital Paris France; ^17^ Departments of Neurology, Radiology and Neuroscience Washington University St. Louis Missouri USA; ^18^ Department of Neurology University of Colorado Aurora Colorado USA; ^19^ Movement Disorders Unit, Department of Neurology Westmead Hospital & Sydney Medical School, University of Sydney Sydney New South Wales Australia; ^20^ Department of Neurology University of Rochester Rochester New York USA; ^21^ Baylor College of Medicine Department of Neurology Houston Texas USA; ^22^ Department of Neurology University of Florida Gainesville Florida USA; ^23^ James J and Joan A Gardner Center for Parkinson's Disease and Movement Disorders University of Cincinnati Cincinnati Ohio USA; ^24^ Department of Psychology University of Memphis, Veracity Neuroscience LLC Memphis Tennessee USA; ^25^ Department of Neurology Virginia Commonwealth University Richmond Virginia USA; ^26^ Neurology University of Maryland School of Medicine Baltimore Maryland USA; ^27^ Department of Neurology Perelman School of Medicine at the University of Pennsylvania Philadelphia Pennsylvania USA; ^28^ Institute of Clinical Molecular Biology Christian‐Albrechts‐Universität and University Hospital Schleswig‐Holstein, Campus Kiel Kiel Germany; ^29^ Department of Neurology University Hospital Schleswig‐Holstein, Campus Lübeck Lübeck Germany

**Keywords:** dystonia, exome sequencing, genetic heterogeneity, pathogenic variants

## Abstract

**Objective:**

Dystonia is one of the most prevalent movement disorders, characterized by significant clinical and etiological heterogeneity. Despite considerable heritability (~25%), the etiology in most patients remains elusive. Moreover, understanding correlations between clinical manifestations and genetic variants has become increasingly complex.

**Methods:**

Exome sequencing was conducted on 1924 genetically unsolved, mainly late‐onset isolated dystonia patients, recruited primarily from two dystonia registries (DysTract and the Dystonia Coalition). Rare variants in genes previously linked to dystonia (*n* = 406) were examined, confirmed via Sanger sequencing, and analyzed for segregation when possible.

**Results:**

We identified 137 distinct likely pathogenic/pathogenic variants (according to ACMG criteria) across 51 genes in 163/1924 patients, including 153/1895 index patients (diagnostic yield 8.1%). The strongest predictors of a genetic diagnosis were generalized dystonia (28.6% yield) and age at onset (20.4% yield in patients with onset < 30 years). Notably, 56.2% of these variants were novel, with recurrent variants in *EIF2AK2*, *VPS16*, *KCNMA1*, and *SLC2A1*. Additionally, 321 index patients (16.9%) harbored variants of uncertain significance in 102 genes. The most frequently implicated genes included *VPS16*, *THAP1*, *GCH1*, *SGCE*, *GNAL*, and *KMT2B.* Presumably pathogenic variants in less well‐established dystonia genes were also found, including *KCNMA1*, *KIF1A*, and *ZMYND11.* At least six variants (in *ADCY5*, *GNB1*, *IR2BPL, KCNN2*, *KMT2B*, and *VPS16*) occurred de novo, supporting pathogenicity.

**Interpretation:**

This study provides valuable insights into the genetic landscape of dystonia, underscores the utility of exome sequencing for diagnosis, substantiates several candidate genes, and expands the phenotypic spectrum of some genes to include prominent, sometimes isolated dystonia.

## 1 Introduction

Dystonia is one of the most prevalent movement disorders, characterized by involuntary muscle contractions leading to abnormal postures and repetitive movements [1]. Its clinical manifestations span a broad spectrum, from isolated cases to those combined with other movement disorders like parkinsonism, myoclonus, or chorea, to patients with additional neurological or systemic features [1].

An essential aspect of establishing a dystonia diagnosis and appropriate treatment lies in genetic testing. However, despite the disease's considerable heritability, with about 25% of patients having affected relatives [2], and the identification of several causal genes, a significant proportion of patients remain genetically unsolved, particularly those with later‐onset focal dystonia—the most common form observed in epidemiological studies [3, 4]. Genetic testing is complicated by the highly heterogeneous nature of the disease [4, 5, 6], the ever‐expanding list of dystonia‐linked genes, and the reliance on clinical diagnosis due to the absence of an established biomarker. Presently, the Movement Disorder Society Task Force for the Nomenclature of Genetic Movement Disorders (MDS Nomenclature Task Force) recognizes 53 established dystonia genes [7]. Additionally, numerous genes have been linked to dystonia as a feature of other neurological disorders. With ongoing sequencing efforts, the list of unconfirmed dystonia candidate genes continues to grow, with many awaiting replication by independent studies [4, 7, 8, 9], altogether resulting in over 400 potential dystonia‐linked genes [10].

The phenotypic presentations of dystonia are diverse, with considerable overlap between the different genetic forms [11, 12]. Instances of classical phenotypes expanding over time or a single gene causing multiple distinct phenotypes (pleiotropy) further highlight this complexity. In this regard, an increasing number of genes, previously associated with other neurological conditions, including developmental delay, epileptic encephalopathy, and ataxia, are now recognized to play a significant role in patients with prominent, sometimes isolated dystonia [8, 13].

As the phenotypic and mutational spectrum of dystonia expands, newly identified genetic variants require thorough evaluation to establish a causal gene‐disease link. Guidelines from the American College of Medical Genetics and Genomics (ACMG) [14], incorporating information from segregation analysis, in silico predictions, and population databases, among others, are instrumental in this task. For some genes, specific functional assays have been developed to assess the impact of variants on protein function, such as aberrant CpG methylation serving as a functional readout for evaluating variants in the dystonia gene *KMT2B* [15]. The most compelling evidence for novel dystonia genes, however, will always come from replicating the findings in independent patients.

Given dystonia's rarity and genetic complexity, large cohorts are indispensable for comprehensively unraveling its genetic spectrum. To achieve this, we performed exome sequencing on 1924 dystonia patients. In contrast to other dystonia cohorts typically enriched with early‐onset, symptom‐complex patients, this cohort is unique in that it predominantly consists of adult‐onset isolated dystonia patients, corresponding to the composition of patients seen in epidemiological studies. We describe detailed phenotypic and genetic findings in over 400 genes previously linked to dystonia, with the aim of facilitating future data aggregation, advancing clinical variant interpretation, improving genotype–phenotype correlations, and contributing to the validation of emerging dystonia genes and their associated phenotypes.

## 2 Methods

### 2.1 Study Population

A total of 1967 samples were included in the exome sequencing study, comprising 1950 dystonia patients and 17 unaffected family members. One sample failed sequencing, and 25 samples were removed after relationship analysis (Methods S1) revealed that those sample IDs referred to identical patients recruited twice from different sites. Altogether, this yielded a total number of 1924 patients with dystonia without a previous genetic diagnosis who were included in the final cohort.

The vast majority of samples were recruited in the framework of two large dystonia registries, DysTract (https://www.isms.uni‐luebeck.de/forschung‐1/deutsches‐dystonie‐register) and the Dystonia Coalition (https://www.dystoniacoalition.org/). These samples had undergone genetic prescreening, comprising: (1) Hot‐spot screening for known pathogenic variants in dystonia genes in about 80% of the samples using the Global Screening Array (GSA V01, Illumina; with custom content) and (2) Gene panel sequencing in about a third of the samples, comprising the genes *ANO3*, *GCH1*, *GNAL*, *KMT2B*, *PRKR*A, *SGCE*, *THAP1*, and *TOR1A* (results published elsewhere [16, 17]). Patients with negative prescreening, early disease onset, positive family history, or multisite involvement were prioritized when selecting samples from the dystonia registries. Patient samples were collected in movement disorder clinics across Europe, North America, and Australia. Additionally, 72 samples from patients of Asian ethnicity (primarily from Malaysia) were included. The total sample consisted of diverse ethnic backgrounds, including White (*n* = 1787), Asian (*n* = 72), African American (*n* = 32), Latino (*n* = 18), Ashkenazi Jewish (*n* = 7), American Indian (*n* = 5), and Mixed (*n* = 3) individuals. Patients were phenotyped by movement disorder specialists, and secondary causes, including acquired dystonia, were excluded. Written informed consent was obtained from all participants prior to genetic testing, and the study was approved by the ethics committee at the University of Lübeck (04‐180).

### 2.2 Sequencing and Data Processing

Genomic DNA was extracted from peripheral blood samples, and exome sequencing was performed at the Competence Centre for Genomic Analysis in Kiel, Germany, using Illumina NovaSeq. Sequencing reads were preprocessed and aligned to the Hg38 reference genome. Variant calling and annotation were conducted using DeepVariant, GLnexus, and VEP, with further details provided in the Methods S1.

### 2.3 Variant Filtering

A comprehensive list of dystonia‐linked genes was generated by systematically querying the PubMed database (search term: dystoni* [TITLE/ABSTRACT] AND (gene* OR genetic* OR mutation* OR mutated OR varia*) AND “english”[Language]) until December 2024, resulting in 406 distinct genes that have been linked to dystonic symptoms in the literature (Table S1). We utilized this phenotype‐driven candidate gene list to identify genetic causes in our dystonia sample.

We filtered out variants with the following criteria: (a) low variant allele frequency (< 20%), (b) low sequencing depth (< 10 reads), (c) synonymous variants, (d) minor allele frequency > 0.05% in the gnomAD population database, (e) variants classified as benign or likely benign in ClinVar, and (f) single heterozygous variants in recessive disease genes. Variants with an allele frequency of 0.25–0.75 were considered heterozygous. For established dystonia genes that have been assigned a DYT prefix by the MDS Nomenclature Task Force [7], the remaining variants underwent detailed individual evaluation using ACMG standards and guidelines [14]. For genes not classified as established dystonia genes, additional filtering was applied before individual evaluation by ACMG standards: excluding single heterozygous loss‐of‐function (LoF) variants in genes with good tolerance for such variants (gnomAD pLI‐score = 0), missense variants classified as likely benign by the alpha missense score [18], and variants with a low CADD Score (< 10) [19].

All likely pathogenic or pathogenic variants were considered disease‐causing and underwent validation through Sanger sequencing. Segregation analysis was performed if family members' DNA was available.

Relevant literature was reviewed to assess phenotypic and genetic information for each gene. Phenotype–genotype relationships were categorized as consistent, partially consistent, quite inconsistent, or unclear/unknown. The unclear/unknown category applied to cases with limited clinical information or candidate genes lacking an established gene‐phenotype relationship.

### 2.4 Statistical Analysis

Statistical analysis and visualization were performed in R version 4.3.2 using base R functions for statistical tests and data manipulation, the pROC package for generating and analyzing ROC curves, and the ggplot2 package for visualizations. For calculating diagnostic yield, patients were grouped based on clinical criteria, including age at onset (AAO: < 30, 30–50, and > 50 years), dystonia subtypes (focal, segmental/multifocal, generalized), family history (positive or negative), and the presence of additional features versus isolated dystonia. These groupings, except AAO, were also used for ROC analysis and statistical comparisons using Fisher's exact test. AAO was treated as a continuous variable for the Mann–Whitney *U* test comparing resolved and unresolved patients and ROC curve analysis. This approach was chosen to capture clinically relevant differences. Missing data were handled by excluding these patients. All statistical tests were two‐sided, and a *p* value < 0.05 was considered statistically significant.

### 2.5 Episignature Analysis for *KMT2B* Variants

For patients with rare variants in *KMT2B* and a sufficient amount of DNA available (*n* = 45), the functional effect was assessed by analyzing the disease‐specific methylation pattern (“episignature”) in peripheral blood, using the Illumina MethylationEPIC BeadChip. The mean of the normalized methylation levels (mean(*z*)) and the coefficient of variation (CV = SD/|mean|) were used as quantifiers (Methods S1).

## 3 Results

### 3.1 Patient Characteristics and Diagnostic Yield

There were 1895 index patients and 29 affected family members among the 1924 included unique dystonia patients. Key patient characteristics are summarized in Table 1. Over half of the patients (54.3%) had focal dystonia, with cervical dystonia being the most common subtype (*n* = 550, 28.6%). About 93% of all patients had isolated dystonia, while in 130 patients (6.8%), additional neurological (other than tremor) or systemic features were reported, such as bradykinesia (*n* = 41), ataxia (*n* = 35), myoclonus (*n* = 35), or neurodevelopmental features (*n* = 12).

**TABLE 1:** Patient characteristics in the investigated dystonia sample by diagnostic outcome.

	All patients	Patients with diagnostic variant	Patients with VUS	Yet‐unsolved patients
Total patients	1924	163	323	1438
Index patients	1895	153 (8.1%)	321 (16.9%)	1421 (75.0%)
Age at examination (median, IQR)	54 years (43–65)	46 years (32.5–59.5)	55 years (45–65)	55 years (44–66)
Age at onset (median, IQR)	33 years (22.5–43.5)	19 years (5.25–32.75)	34 years (24–44)	34 years (24–44)
Sex				
Males	776 (40.3%)	70 (42.9%)	121 (37.5%)	585 (40.7%)
Females	1148 (59.7%)	93 (57.1%)	202 (65.5%)	853 (59.3%)
Family history^a^				
Positive	561 (29.6%)	51 (33.3%)	73 (22.7%)	437 (30.9%)
Negative	1233 (65.1%)	93 (60.8%)	187 (58.3%)	953 (66.9%)
NA	101 (5.3%)	9 (5.9%)	61 (19.0%)	31 (2.2%)
Type of dystonia				
Generalized	236 (12.3%)	73 (44.8%)	30 (9.3%)	133 (9.2%)
Segmental/multifocal	549 (28.5%)	36 (22.1%)	92 (28.5%)	421 (29.3%)
Focal	1045 (54.3%)	53 (32.5%)	193 (59.8%)	799 (55.6%)
NA	94 (4.9%)	1 (0.6%)	8 (2.5%)	85 (5.9%)
Isolated dystonia	1794 (93.2%)	112 (68.7%)	308 (95.4%)	1374 (95.5%)
Additional neurological features reported (other than tremor)	130 (6.8%)	51 (31.3%)	15 (4.6%)	64 (4.5%)

Abbreviations: IQR, interquartile range; NA, information not available; VUS, variant of uncertain significance.

^a^ Calculated for index patients only.

**FIGURE 1:**
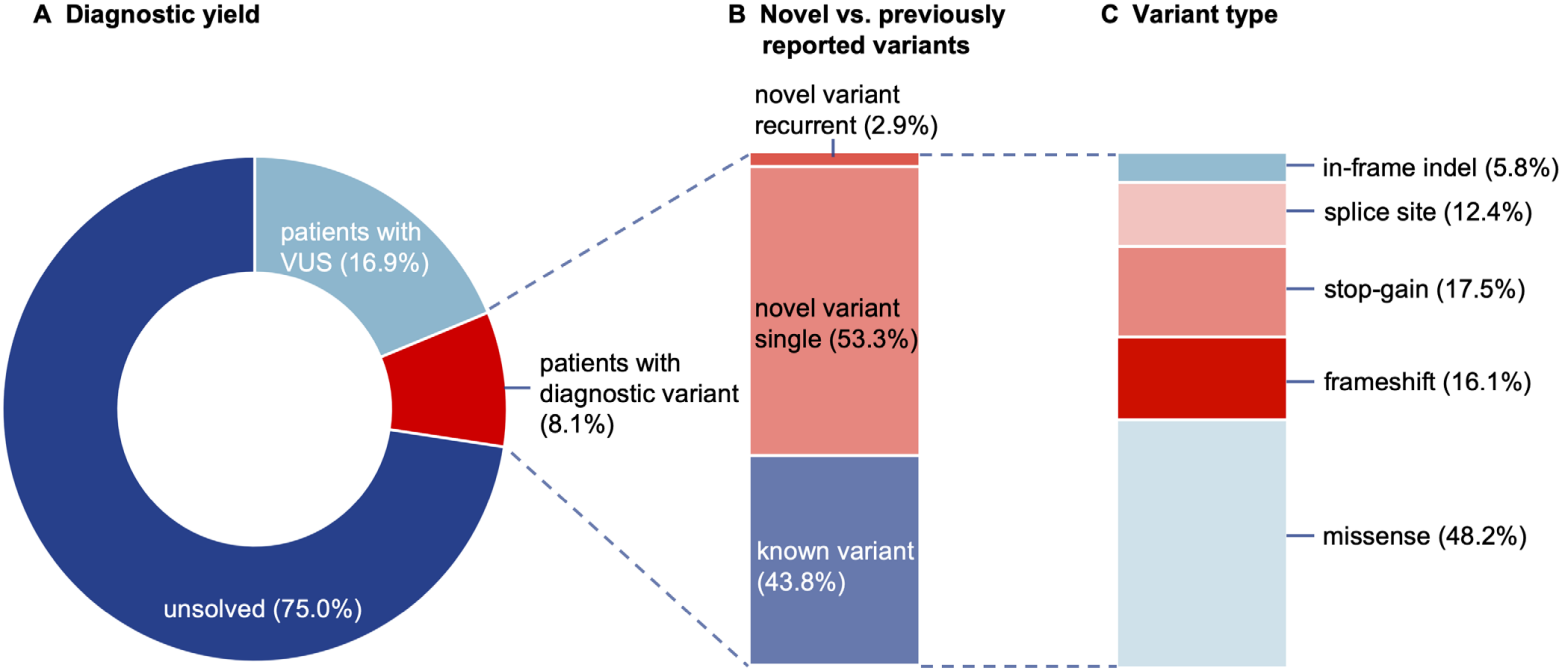
Overview of exome sequencing results. (A) Proportion of index patients for whom a diagnostic variant was identified, who carry a variant of uncertain significance (VUS), and who remain unsolved after searching for pathogenic variants in dystonia‐linked genes. (B) Proportion of diagnostic variants that were previously reported (known variant), not previously reported and detected in a single patient or pedigree (novel variant single), and not previously reported and found recurrently in at least two unrelated dystonia patients (novel variant recurrent). (C) Distribution of diagnostic variants by variant type.

**FIGURE 2:**
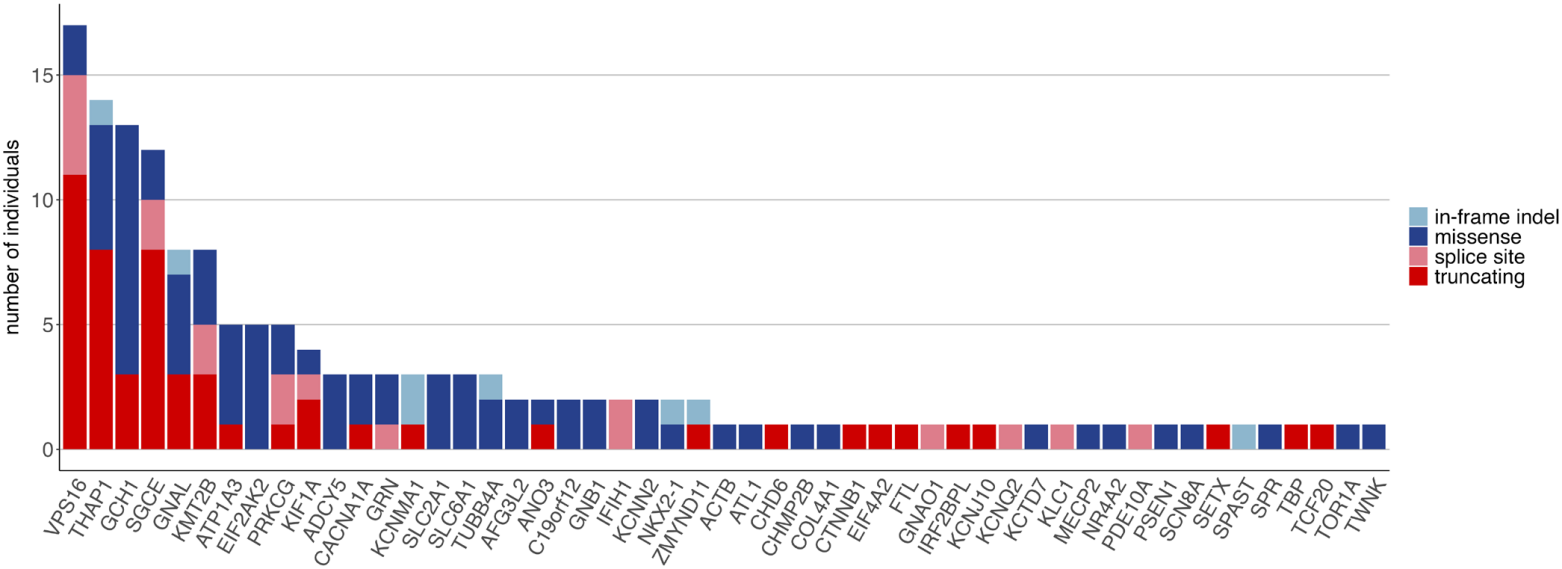
Genetic landscape in our dystonia sample (*n* = 1895 index patients) identified by exome sequencing. The number of individuals harboring a presumably pathogenic variant in genes previously linked to dystonia is shown, representing 51 distinct genetic forms and a total diagnostic yield of 8.1%. Truncating variants include stop‐gain and frameshift variants.

**FIGURE 3:**
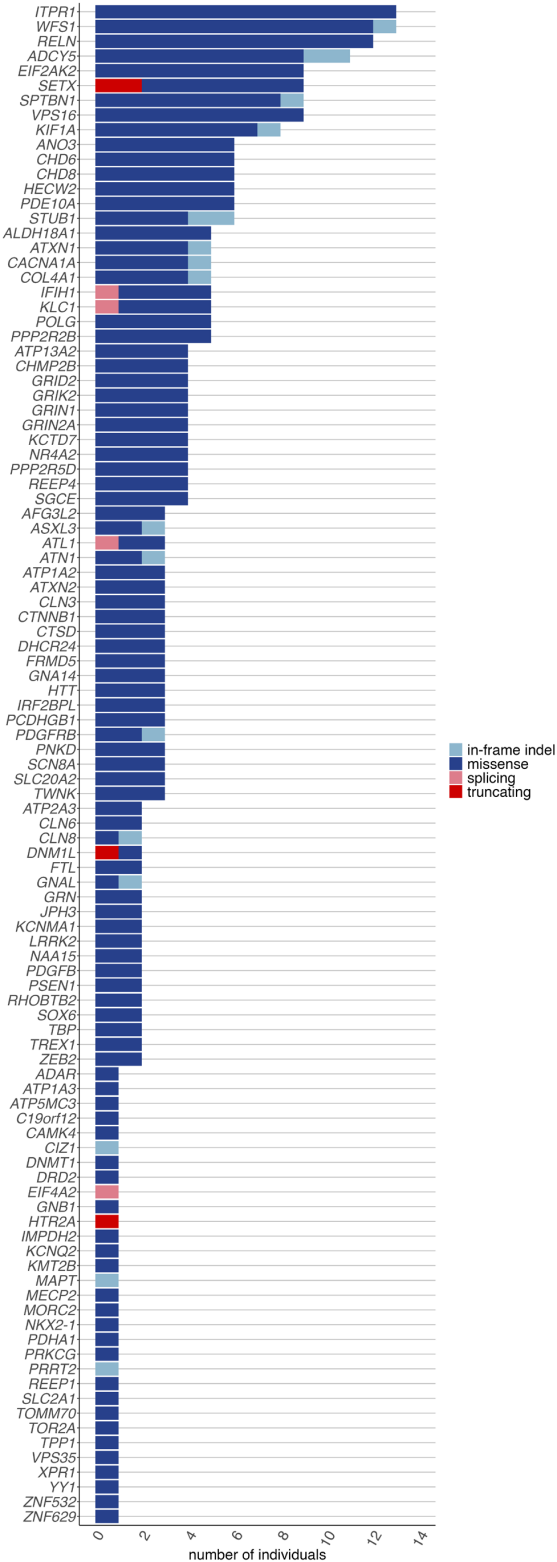
Variants of uncertain significance in our dystonia sample (*n* = 1895 index patients) identified by exome sequencing. The number of individuals harboring variants of uncertain significance in genes previously linked to dystonia is shown. This represents 329 distinct variants in 102 genes found in 321 index patients (16.9%). Truncating variants include stop‐gain and frameshift variants.

Exome sequencing revealed likely pathogenic or pathogenic variants in dystonia‐linked genes in 163 patients, including 153 out of 1895 index patients (8.1% diagnostic yield), with an additional 321 index patients (16.9%) having variants of uncertain significance (VUS) in dystonia‐linked genes (Figures 1, 2, 3, Tables S2 and S3).

Compared to unsolved patients and those with VUS combined, patients with a genetic diagnosis had a significantly earlier AAO (Mann–Whitney *U* test, *p* < 0.0001), higher prevalence of generalized dystonia (Fisher's exact test, *p* < 0.0001), positive family history (Fisher's exact test, *p* = 0.043), and additional clinical features (Fisher's exact test, *p* < 0.0001). There were no significant differences between patients with VUS and unsolved patients regarding these characteristics.

**FIGURE 4:**
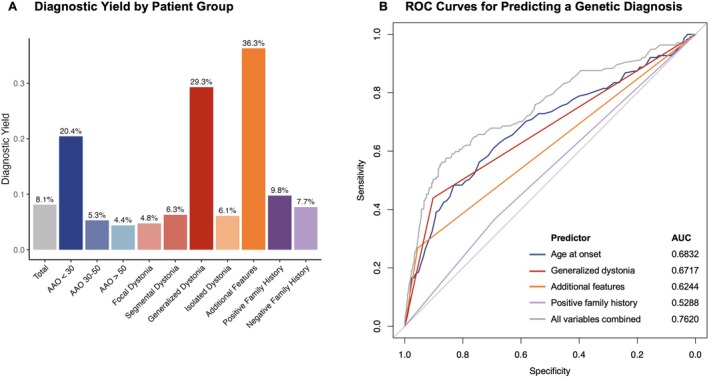
Diagnostic yield across patient groups and predictive performance of clinical factors for genetic diagnosis. (A) Bar plot showing the diagnostic yield (%) across different patient groups, considering index patients only. The segmental dystonia group includes patients with multifocal dystonia. (B) ROC curves illustrating the performance of individual predictors and a combined model for genetic diagnosis. The predictors include age at onset, generalized dystonia, additional features, positive family history, and a combined model incorporating all four predictors. Each curve shows the balance between sensitivity (true positive rate) and 1‐specificity (false positive rate), with the Area Under the Curve (AUC) indicating predictive performance.

The diagnostic yield varied substantially across index patient groups, with the highest yield in patients with additional features (36.3%), followed by those with generalized dystonia (29.3%) and those with an AAO < 30 years (20.4%) (Figure 4a). ROC curves were generated to evaluate the predictive performance of four variables—AAO, positive family history, generalized dystonia, and additional features—along with a combined model incorporating all four variables (Figure 4b). The area under the curve (AUC), which measures predictive accuracy, was 0.68 for AAO, 0.67 for generalized dystonia, 0.62 for additional features, and 0.53 for family history. The combined model, incorporating all four predictors, achieved an AUC of 0.76, indicating the best predictive performance, with AAO and generalized dystonia being the strongest individual predictors.

### 3.2 Genetic Findings

Among the index patients with a genetic diagnosis (*n* = 153, 8.1%), 137 distinct variants were identified in 51 genes previously linked to dystonic syndromes (Table S2, Figure 2). The majority (131/137, 95.6%) of variants were found in the heterozygous state in genes known to be linked to dominant inheritance. One variant in *SLC6A1* was identified in the mosaic state (variant allele frequency = 0.2 and clearly reduced peak in Sanger sequences). One variant (in *MECP2*) was found in the hemizygous state in a male patient, associated with X‐linked inheritance, and four variants (two in *GCH1* and one each in *SETX* and *SPR*) were biallelic and linked to autosomal recessive disorders. At least six variants occurred de novo (in *ADCY5*, *GNB1*, *IR2BPL, KCNN2*, *KMT2B*, and *VPS16*) (Figure S1). Over half of the identified presumably pathogenic variants (77/137; 56.2%) were not previously reported in ClinVar or MDSGene [20]. Among these, four recurred in at least two unrelated index patients (in *EIF2AK2*, *KCNMA1*, *SLC2A1*, and *VPS16*; see below), supporting pathogenicity. Details about the variants, pathogenicity scoring, and clinical information can be found in Table S2.

A wide spectrum of genetic causes was identified, encompassing established dystonia genes as defined by the MDS Nomenclature Task Force in 103 out of 163 diagnoses, movement disorder or developmental delay genes that frequently include dystonia as a phenotype (9/163 and 4/163 diagnoses, respectively), genes typically linked to other neurological disorders in which dystonia has been rarely reported (41/163 diagnoses), and dystonia candidate genes (7/163 diagnoses) (Table S2). Genotype–phenotype relationships were consistent with the literature in most patients (111/163; 68.1%), partially consistent in 14 (8.6%), quite inconsistent in 25 (15.3%), and unclear/unknown in 14 (8.6%) (Table S2). Notably, all variants in established dystonia genes demonstrated at least partial genotype–phenotype consistency. Meanwhile, cases classified as “quite inconsistent” were mostly focal (*n* = 15) or segmental/multifocal (*n* = 8) dystonia and involved variants in genes typically associated with other disorders where dystonia is rarely reported, such as *C19orf12*, *GRN*, *KIF1A*, and *PRKCG*.

### 3.3 Findings in Established Dystonia Genes

The most frequently implicated genes included the well‐established isolated and combined dystonia genes *VPS16* (*n* = 17 index patients), *THAP1* (*n* = 14), *GCH1* (*n* = 13), *SGCE* (*n* = 12), *GNAL* (*n* = 8), and *KMT2B* (*n* = 8). Other dystonia genes with likely pathogenic or pathogenic variants were *EIF2AK2* (*n* = 5 index patients), *ANO3* (*n* = 2), *EIF4A2* (*n* = 1), and *TOR1A* (*n* = 1) for genes causing isolated dystonia. Of these, the genes *VPS16*, *EIF2AK2*, and *EIF4A2*, which were relatively recently associated with dystonia, were not included in the prescreening process. The frequencies of these genetic forms in our dystonia sample were 0.9% (17/1895 index patients) for *VPS16*, 0.3% (5/1895) for *EIF2AK2*, and 0.05% (1/1895) for *EIF4A2*. Additionally, likely pathogenic or pathogenic variants were found in *ATP1A3* (*n* = 5) and *GNAO1* (*n* = 1) for genes causing combined dystonia, as well as *ACTB* (*n* = 1), *IRF2BPL* (*n* = 1), *SPR* (*n* = 1), and *TUBB4A* (*n* = 3) for genes linked to dystonia with other neurological or systemic features (Table S2). Notably, 43 out of 84 variants (51.2%) in established dystonia genes were not previously reported, including two novel recurrent variants (*EIF2AK2*:p.Pro31Ser and *VPS16*:p.Ile484Thrfs*70).

For six variants, segregation analysis revealed that the identified variant co‐segregated with the dystonia phenotype in the affected families, supporting its pathogenic role (*EIF2AK2*:p.Pro31Ser, *EIF2AK2*:p.Gly130Arg, *THAP1*:p.Ser51Arg, *GCH1*:p.Glu61*, *SGCE*:p.Met174Lys, *SGCE*:c.233‐1G>A:p.?) (Figure S1). Additionally, three variants were confirmed to be de novo (*KMT2B*:p.Arg1771Trp, *VPS16*:p.Lys397Glu, and *IRF2BPL*:p.Gln167*).

Of note, for several established dystonia genes, no carrier of a disease‐causing variant was identified in our large patient group (e.g., in *AOPEP*, *HPCA*, *PRKRA*, and *TH*).

Interestingly, one patient with early‐onset generalized dystonia harbored two known likely pathogenic variants in different genes (*TOR1A*:p.Arg288Gln and *SPAST*:p.Glu418del).

For 32 rare variants in *KMT2B*, identified in 45 patients, the disease‐specific methylation pattern (“episignature”) in patients' blood was analyzed to assess their functional effect. Two of the tested variants were shown to result in strong hypermethylation and showed mean(*z*) and CV values characteristic of loss of KMT2B function (Table S4), which were interpreted as positive functional evidence during pathogenicity scoring. Notably, these two variants were canonical splice site variants (c.4779+1G>A and c.3789+1G>A), not previously reported, and associated with childhood‐onset generalized dystonia (Table S2). Family history was negative in both patients; however, family members were not available to test whether the variants arose de novo.

### 3.4 Findings in Movement Disorder Genes That Frequently Include Dystonia

Variants in movement disorder genes frequently comprising dystonia as a phenotype included recurrent variants in *ADCY5*, *C19orf12*, and *SLC2A1*. Two unrelated patients with early‐onset mixed movement disorders, including generalized dystonia, carried the same pathogenic variant in *ADCY5* (p.Arg418Trp), proven de novo in one case. A known missense variant in *C19orf12* (p.Gly58Arg) was found in two unrelated patients with adult‐onset focal dystonia (cervical dystonia and blepharospasm), both without additional features. Two unrelated patients with adult‐onset cervical dystonia, one also involving the upper limbs and shoulders, carried a novel *SLC2A1* variant (p.Gln25Lys). Additionally, a known pathogenic variant in *FTL* (p.Glu58*) was identified in a patient with isolated adult‐onset cervical dystonia.

### 3.5 Findings in Neurodevelopmental Delay Genes That Frequently Include Dystonia

Presumably pathogenic variants in neurodevelopmental disorder genes that frequently include dystonia as a phenotype comprised a novel nonsense variant in *CTNNB1* (p.Gln4*) in a patient with isolated cervical dystonia, a recurrent de novo missense variant in *GNB1* (p.Lys337Gln) in two unrelated patients with infancy‐onset generalized dystonia—one of whom had a complex phenotype including global developmental delay, hypotonia, myoclonus, and vertical supranuclear gaze palsy, and was recently published [21]—and a known hemizygous missense variant in *MECP2* (p.Arg97Cys) in a male patient with cervical and truncal dystonia, along with developmental delay (Table S2).

### 3.6 Findings in Genes Usually Linked to Non‐Dystonia Phenotypes

We identified 41 dystonia patients with likely pathogenic or pathogenic variants in 23 uncommonly dystonia‐linked genes. Of these, seven showed consistent genotype–phenotype relationships, four showed partial consistency, 21 were quite inconsistent, and nine were unclear/unknown (Table S2). While 13 variants had been reported in the context of other phenotypes, 24 were previously undescribed.

Genes with pathogenic variants in several affected individuals included genes usually associated with spinocerebellar ataxia (*PRKCG* [*n* = 5], *CACNA1A* [*n* = 3], and *AFG3L2* [*n* = 2]), spastic paraplegia (*KIF1A* [*n* = 4]), frontotemporal dementia (*GRN* [*n* = 3]), paroxysmal movement disorders (*KCNMA1* [*n* = 3]), and others (*SLC6A1* [*n* = 3], *IFIH1* [*n* = 2], and *NKX2‐1* [*n* = 2]), while only one carrier of a presumably pathogenic variant was found in the remaining 14 uncommon dystonia genes (in *ATL1*, *CHMP2B*, *COL4A1*, *KCNJ10*, *KCNQ2*, *KCTD7*, *PDE10*, *PSEN1*, *SCN8A*, *SETX*, *SPAST*, *TBP*, *TCF20*, and *TWNK*).

Notably, four variants were recurrently found in at least two unrelated patients (*AFG3L2*:p.Arg280Trp, *GRN*:p.Cys139Arg, *IFIH1*:c.454‐1G>T, *KCNMA1*: p.Ser11_Ser12delinsGly). Except for one patient with the *AFG3L2* variant, these showed quite inconsistent or unclear genotype–phenotype relationships; that is, in our study, they were identified in patients with isolated, persistent dystonia, but are otherwise usually reported in conjunction with other phenotypes.

### 3.7 Findings in Dystonia Candidate Genes

We also investigated dystonia candidate genes, uncovering seven novel likely pathogenic variants that were all absent from control databases and predicted to be deleterious (Table S2). This included two missense variants in *KCNN2*, with one (p.Leu611Ile) occurring de novo in a patient with infancy‐onset generalized myoclonus‐dystonia who also showed signs of bradykinesia and ataxia.

Two variants were identified in *ZMYND11*, including a stop‐gain variant (p.Arg495*) in a patient with infancy‐onset generalized dystonia and an in‐frame insertion (p.His315dup) in a patient with cervical dystonia, tremor, and polyneuropathy with unknown AAO. One variant each was identified in the genes *CHD6* (p.Glu2697Thrfs*29 in a patient with adolescence‐onset generalized dystonia with myoclonus and ataxia), *KLC1* (c.*1+1G>A in a patient with adult‐onset cervical dystonia), and *NR4A2* (p.Gln273Arg in a patient with adult‐onset cervical dystonia). For *KLC1* and *NR4A2*, five and four additional patients, respectively, with similar phenotypes of adult‐onset focal dystonia, carried VUS (Table S3, Figure 3).

No carriers of presumably pathogenic variants in other dystonia candidate genes were detected among our 1924 patients, including the recently proposed genes *ATP5F1B*, *ACBD6*, and *SPTBN1*. However, several VUS were identified in candidate genes, including *SPTBN1*, where nine patients were found to carry rare missense and in‐frame indels. They all shared an adult‐onset focal or segmental dystonia phenotype involving the upper body (Table S3).

While the clinical relevance of the identified VUS remains uncertain, the observed clustering of rare variants in these genes among patients with similar phenotypes may warrant further investigation.

## 4 Discussion

This study comprehensively examined the genetic landscape of dystonia in 1924 patients, the most extensive next‐generation sequencing study conducted in dystonia patients to date. Importantly, this sample predominantly consists of adult‐onset isolated dystonia patients, the most frequent but yet understudied form of dystonia. Despite negative prescreening for many known pathogenic variants, the diagnostic yield of the present study was 8.1%, with an additional 16.9% of index patients harboring VUS. Patients with diagnostic variants had a significantly lower median AAO and a higher prevalence of generalized dystonia, additional neurological or systemic features, and positive family history compared to those without a diagnosis. ROC curve analysis indicated that AAO and the presence of generalized dystonia were the strongest predictors of a genetic diagnosis, with diagnostic yields of 28.6% in generalized dystonia and 20.4% in AAO < 30. These findings align with prior studies and highlight the importance of considering exome sequencing in these patient groups [4, 22]. Diagnostic yields in dystonia vary widely based on patient selection, ranging from 11.7% to 37.5% [23]. For instance, one study in 764 dystonia patients reported a 19% yield overall; however, the yield reached almost 50% in patients with early‐onset (< 20 years), generalized, non‐isolated dystonia, while it was as low as 1% in patients with late‐onset, isolated, focal dystonia [4].

Our findings provide crucial insights into the relevance of genetic forms of dystonia and their corresponding phenotypes and will aid future variant interpretation and clinical diagnostics. Presumably pathogenic variants include 77 (56.2%) novel variants, with recurrent variants in *EIF2AK2*, *VPS16*, *KCNMA1*, and *SLC2A1*, and novel variant types such as two splice site variants in *KMT2B*. For *KMT2B*, we conducted episignature analysis, which has proven to reliably distinguish between benign and pathogenic variants [15]. An overview of the results of episignature testing has also been added to the MDSGene website (https://www.mdsgene.org).

### 4.1 Genotype–Phenotype Relationships and Phenotypic Expansions

This study identified diverse genetic causes of dystonia, with presumably pathogenic variants in 51 distinct genes, highlighting the genetic heterogeneity of dystonia. Most detected variants aligned with the phenotypic spectrum reported in the literature (Table S2), though some unusual findings were also observed (Table S2, Discussion S1). As new discoveries unfold, understanding the correlations between clinical manifestations and genetic variants has become increasingly complex due to mechanisms such as variable expressivity, incomplete penetrance, and genetic pleiotropy. Indeed, our study revealed instances where the genotype–phenotype correlation was inconsistent with previous reports. This included variants in *AFG3L2*, *ATL1*, *C19orf12*, *CHMP2B*, *COL4A1*, *GRN*, *KCNMA1*, *KCTD7*, *KIF1A*, *PDE10A*, *PRKCG*, *PSEN1*, *SCN8A*, *SLC2A1*, and *SLC6A1*. For some of these, further investigations are warranted to evaluate their true pathogenicity.

Noteworthy are three patients with *GRN* variants which were previously described in frontotemporal dementia patients [24, 25]. However, all our patients had adult‐onset focal dystonia (cervical or upper limb) without additional features (AAE: 25–76 years). This suggests that dystonia may occur without or prior to cognitive and behavioral symptoms due to *GRN* variants.

Further, we identified three patients with novel variants in *KCNMA1*, including a recurrent in‐frame deletion and a frameshift variant, all absent in gnomAD v4. *KCNMA1* is typically associated with paroxysmal non‐kinesigenic dyskinesia, developmental delay, and seizures, with LoF being a known disease mechanism [26]. Our patients presented with childhood‐ or adult‐onset focal persistent dystonia (cervical or upper limb) without additional features, except for tremor and left eye ophthalmoplegia in one patient. Although movement disorders are common in *KCNMA1*‐related disease, particularly in LoF variants [26], patients with isolated dystonia have not yet been reported.

We also identified four likely pathogenic variants in *KIF1A*: two truncating, one splice site, and one missense variant affecting the kinesin motor domain, where the majority of pathogenic variants are found [27]. *KIF1A* is typically associated with spastic paraplegia, but our patients presented with isolated upper body dystonia (cranial, cervical, or upper limbs), with two patients exhibiting tremor and one muscle atrophy at the shoulders. While dystonia is not uncommon in *KIF1A*‐related diseases, this is the first report of patients without spasticity, potentially expanding the phenotypic spectrum.

Another interesting gene was *CACNA1A*, which has been linked to several diseases, including episodic ataxia, cerebellar ataxia, and developmental and epileptic encephalopathy. Recent reports have shown that dystonia can also be primary and/or generalized in cases with *CACNA1A* variants [28, 29, 30]. In our study, we identified three patients with likely pathogenic variants, all having isolated dystonia affecting cranial or cervical muscles, with disease onset in their 40s. This confirms that variants in *CACNA1A* are also linked to isolated dystonia.

### 4.2 Dystonia Candidate Genes

Screening our data for proposed dystonia candidate genes identified presumably pathogenic variants in *CHD6*, *KCNN2*, *KLC1*, *NR4A2*, and *ZMYND11*, but not in others, for example, *ATP5F1B*, *ACBD6*, *CIZ1*, *SHQ1*, and *SPTBN1*.

Variants in *KCNN2* were initially linked to neurodevelopmental disorders, sometimes with movement disorders [31], and later associated with tremulous myoclonus‐dystonia [32], We identified two patients with likely pathogenic missense variants. The first had infancy‐onset generalized dystonia, myoclonus, ataxia, and bradykinesia—symptoms previously reported in *KCNN2*‐related disease [31]—and carried a de novo variant, supporting its pathogenicity and confirming its role in myoclonus‐dystonia. The second variant (p.Ser376Leu), located in the protein's highly missense‐intolerant ion channel domain [33] which harbors other pathogenic variants [31, 32], was found in a patient with isolated cervical dystonia since age 27 years, potentially broadening the phenotypic spectrum of *KCNN2*.

In 2020, *CHD6*, *KLC1*, and *ZMYND11* were linked to early‐onset dystonia combined with developmental delay or intellectual disability [4]. Here, we identified variants in these genes with different phenotypes. This included a novel frameshift variant in *CHD6* in a patient with adolescence‐onset generalized dystonia and myoclonus, as well as a novel splice‐site variant in *KLC1* in a patient with late‐onset cervical dystonia without additional features. For *ZMYND11*, we observed a stop‐gain variant in a patient with infancy‐onset isolated generalized dystonia and a single amino acid duplication in a patient with cervical dystonia, tremor, and polyneuropathy (AAO unknown).

Although no pathogenic variants were identified in the recently proposed candidate gene *SPTBN1*, we discovered nine patients with rare missense or in‐frame VUS. All these carriers had adolescence or adult onset (median AAO: 40 years, range: 16–46) focal or segmental dystonia, predominantly affecting the neck or upper extremities. Notably, the initial report described a splice site variant in a patient with adolescent‐onset segmental dystonia and developmental delay [4].

Furthermore, we identified a novel, likely pathogenic missense variant in *NR4A2*, a gene recently associated with levodopa‐responsive dystonia and developmental delay [34, 35]. Our patient presented with cervical dystonia with no additional features reported, and information on levodopa responsiveness was unavailable. Additionally, four patients harbored missense variants in *NR4A2* classified as VUS, all located within the ligand‐binding domain at missense‐intolerant locations [33]. These patients all presented with adult‐onset focal dystonia affecting the neck or upper limbs, with AAO ranging from 27 to 44 years.

Notably, our data support a role of pathogenic variants in *CHD6*, *KLC1*, *NR4A2*, and *ZMYND11* in dystonia even without neurodevelopmental features, while the relevance of identified VUS in several candidate genes remains unclear.

### 4.3 Limitations

Our study has some limitations. First, exome sequencing may miss non‐coding variants and structural variations. Additional genome sequencing might increase the diagnostic yield by 5%–10% [36]. Second, the exclusion of patients with known pathogenic variants likely biased the sample towards rarer or novel genetic causes, limiting the generalizability of prevalence and observed genotype–phenotype relationships. This is underlined by the absence of the most frequent dystonia variant, p.E303del in *TOR1A*, which was excluded by prescreening. Third, the classification of VUS remains challenging, and such variants should be treated with caution until further supporting evidence, such as segregation or functional data, becomes available. Access to family members for segregation analysis is often limited, and functional studies are currently available only for a small subset of genes, including *KMT2B*. Additionally, clinical phenotyping has inherent limitations: variability in expert assessments can lead to misdiagnosis, and within this large cohort, some patients may have alternative explanations for their symptoms. While AI‐guided tools like video analysis or machine‐learning diagnostics may reduce this variability in the future, they are not yet implemented in clinical practice. Lastly, missing clinical information is a challenge. Despite recontacting the referring clinicians for all patients with presumably pathogenic variants to review the phenotype and family history, some data was unavailable (indicated as “not mentioned” under additional symptoms in Table S2). This is especially a caveat for variants in genes not typically linked to dystonia and inconsistent phenotype.

## 5 Conclusion and Outlook

Taken together, our study demonstrates the usefulness of exome sequencing to elucidate the molecular basis in a heterogeneous disorder like dystonia. Given the large number of genes and variants linked to dystonia, diagnostic gene‐specific approaches or panels are often impractical, making comprehensive screening methods like exome or genome sequencing the most efficient path to diagnosis. These unbiased screening strategies can be reanalyzed at any time to include novel disease genes, increasing the diagnostic yield in the long term [37]. To aid future variant interpretation, we therefore also provide a list of all (*n* = 321) index patients with VUS in dystonia‐linked genes (Table S3), which may be reclassified as likely pathogenic as more carriers are identified. This is particularly relevant for candidate genes like *CHD6*, *KLC1, NR4A2*, and *SPTBN1*, in which several VUS were identified. Furthermore, this exome data set can be utilized for novel disease gene discovery through gene burden analysis and exome‐wide filtering of single patients or families for deleterious variants.

Finding a genetic diagnosis is invaluable for the patients and their families as it facilitates clinical management, treatment decisions, and genetic counseling, provides prognostic information, and offers crucial insights for the development of targeted therapies.

## Author Contributions

C.Kl., H.B., and K.L. contributed to the conception and design of the study. All authors contributed to the acquisition and/or analysis of the data. M.T. and K.L. contributed to drafting the text or preparing the figures.

## Conflicts of Interest

The authors declare no conflicts of interest.

## Supporting information


Data S1.

Data S2.

## Data Availability

Raw data were generated at the Competence Centre for Genomic Analysis in Kiel, Germany, and are available from the corresponding author on request.
