# Supplementary material for: Genetic Diversity and Expanded Phenotypes in Dystonia: Insights From Large‐Scale Exome Sequencing
Source: Ann Clin Transl Neurol. 2025 Jun 18;12(8):1648–59. doi: 10.1002/acn3.70100 (PMC12343315; doi:10.1002/acn3.70100)
Supplement: Supplementary file 1 — Data S1. [file ACN3-12-1648-s001.pdf]

Supplementary Material

Genetic Diversity and Expanded Phenotypes in Dystonia: Insights from Large-Scale Exome Sequencing

Mirja Thomsen, Fabian Ott, Sebastian Loens, Gamze Kilic-Berkmen, Ai Huey Tan, Shen-Yang Lim, Ebba Lohmann, Kaja M. Schröder, Lea Ipsen, Lena A. Nothacker, Linn Welzel, Alexandra S. Rudnik, Frauke Hinrichs, Thorsten Odorfer, Kirsten E. Zeuner, Friederike Schumann, Andrea A. Kühn, Simone Zittel, Marius Moeller, Robert Pfister, Christoph Kamm, Anthony E. Lang, Yi Wen Tay, Marie Vidailhet, Emmanuel Roze, Joel S. Perlmutter, Jeanne S. Feuerstein, Victor S. C. Fung, Florence Chang, Richard L. Barbano, Steven Bellows, Aparna A. Wagle Shukla, Alberto J. Espay, Mark S. LeDoux, Brian D. Berman, Stephen Reich, Andres Deik, Andre Franke, Michael Wittig, Sören Franzenburg, Jens Volkmann, Norbert Brüggemann, H. A. Jinnah, Tobias Bäumer, Christine Klein, Hauke Busch, Katja Lohmann

Table of Contents

Supplementary Methods	1-2
Supplementary Discussion	2-3
Supplementary Figure S1	4
Supplementary References	5-6
Supplementary Tables 1-4	Excel File

Supplementary Methods

Exome sequencing and data processing

Genomic DNA was extracted from peripheral blood samples using the QIAamp DNA Mini Kit (Qiagen, Hilden, Germany) according to the manufacturer's protocols. The whole exome library preparation and sequencing were conducted at the Competence Centre for Genomic Analysis in Kiel, Germany, utilizing the Illumina DNA Prep with Enrichment kit (Illumina, San Diego, USA) and the IDT xGen Exome v2 baits (Integrated DNA Technologies Coralville, Iowa, USA). Sequencing was performed on an Illumina NovaSeq 6000 instrument using the S4 Flowcell and 150 bp paired-end sequencing with an approximate mean sequencing depth of 150x. Sequenced fastq reads underwent preprocessing to ensure data quality and alignment accuracy. Reads were trimmed, removing reads with fewer than 50 bases, those with excessively high CG content, and low-quality reads with a Phred score <15. Alignment to the Hg38 reference genome was performed using BWA-mem2.[1] Post-alignment processing included Picard tools for read cleaning, sorting, and duplicate marking (CleanSam, SortSam, MarkDuplicates). Mate-pair information was verified with FixMateInformation, reads were assigned to read groups with AddOrReplaceReadGroups, and reads were reordered with ReorderSam. Base quality recalibration was conducted using GATK's Base Quality Recalibration tool, utilizing the Homo_sapiens_assembly38.dbsnp138.vcf from GATK.[2]
Variant calling was performed to identify small insertions or deletions (indels) and single nucleotide variants (SNVs). DeepVariant[3] version 1.2.0 was utilized for variant calling, and GLnexus was employed to merge Variant Calling Files (VCF) into a single cohort file. Variant Calling Files (VCFs) were annotated using VEP v.103. Additional annotation information was provided, including CADD[4] (v1.5) score and annotation from gnomAD[5] exome (r2.1.1) and genome (r.3.0). Sample files were restructured using maftools for efficient handling and analysis.

Relatedness analysis

To identify possible duplicates and related individuals, a two-step approach was employed utilizing GRAPE[6] and NgsRelate.[7]
First, the GRAPE toolkit was used to analyze vcf files from all individuals, identifying IBD segments with the IBIS[8] algorithm and estimating relationship degrees with ERSA.[9] Pairs of individuals estimated to be related to the second degree or closer, along with a custom list of suspected relations based on clinical data or shared rare variants, advanced to the second step.
Second, BAM files were preprocessed to generate allele frequencies and genotype likelihoods using ANGSD.[10] These genotype likelihoods were compared to determine a more accurate percentage of IBD-sharing, yielding kinship coefficients and pairwise relatedness. IBS patterns were also analyzed with KING[11] to obtain R1 and R0 ratios. Individuals were categorized into zero-degree (monozygotic twins or same individual) or first-degree (parent-offspring or full siblings) relationships, and those with implausible R1 and R0 values were filtered out.

Episignature analysis for *KMT2B* variants

To assess the functional effect of *KMT2B* variants, the DYT-KMT2B-specific methylation pattern ("episignature"), comprising 113 specific CpG sites, was analyzed as described.[12] For

this, peripheral blood methylation analysis was performed using the Illumina MethylationEPIC BeadChip.
DNA methylation (DNAm) profiling was conducted using the "Infinium MethylationEPIC" array (Illumina, Inc.). DNA extracts were diluted to approximately 50 ng/µl concentration and then subjected to bisulfite conversion with the EZ DNA Methylation Kit (Zymo Research), following the supplier's alternative incubation conditions for the Illumina Infinium MethylationEPIC Array. The converted DNA samples were then hybridized to the EPIC array and scanned on an iScan instrument (Illumina, Inc.) as per the manufacturer's instructions (Document #1000000077299v0). The raw DNAm intensities were generated using the iScan control software (v2.3.0.0; Illumina, Inc.) and exported in .idat format for further processing and analysis.
Methylation intensities were analyzed using R version 4.2.2 and the minfi package (v1.44). We identified poor-performing probes by minfi::detP and only kept samples that have a mean p-value over all probes <0.05. Stratified quantile normalization was performed for normalization. Following best practices, the dataset was cleaned by removing several poor-performing probes: Probes with detection p-value <0.01 in over 50% of samples, probes with multiple binding sites on the array, probes that multimap on different genetic regions based on bowtie2, and unreliable probes (based on[13]). During data cleaning, 10 of the 113 *KMT2B*-associated probes were filtered out (cg20042692, cg27519958, cg09512891, cg23793686, cg07579404, cg09423283, cg08894761, cg13876206, cg02413092, cg04988061).
The methylation level at each site was assessed as an M-value, which indicates the log2 ratio of the intensities of methylated and unmethylated states. Normalized methylation levels (z-values) for each CpG site in each *KMT2B*-variant carrier were derived by taking the aberration of the M-value from the mean M-value of that site in controls and dividing it by the standard deviation (SD) at that site in controls. As a control group, we used 17 DYT-SGCE patients and repeated the calculation using 38 unaffected individuals to compare the outcomes. We also repeated the calculation using all 113 CpG sites. The mean of the normalized methylation levels (mean(z)) and the coefficient of variation (CV = SD/ |mean|) were calculated for each individual.

Supplementary Discussion

Genotype-phenotype relationships and uncommon findings in established dystonia genes

Most detected variants in established dystonia genes aligned with the phenotypic spectrum reported in the literature (Supplementary Table 2), though some unusual findings were also observed (Supplementary Table 2): One patient with DYT-EIF2AK2 featured bradykinesia and rigidity, symptoms not previously reported in this form.[14,15] A patient with DYT-ANO3 presented with spasticity and parkinsonism, features described only twice in the literature,[14,16] suggesting they may be recurrent characteristics. Bradykinesia and resting tremor were observed in a DYT-VPS16 patient; while resting tremor has been previously reported in one patient,[17] presence of bradykinesia is novel. Additionally, neurodevelopmental delay was noted in another DYT-VPS16 patient, which had not been previously described. Furthermore, chorea was observed in a DYT-VPS16 patient, a feature recently reported in a single patient,[18]

highlighting that additional features are not uncommon in this genetic form despite isolated dystonia cases predominating. We also identified two DYT-VPS16 patients with unusually late ages at onset (64 and 70 years). The reported onset of DYT-VPS16 ranges from 3 to 50 years.[14] The occurrence of both very early-onset and these exceptionally late-onset cases, both caused by truncating variants, suggests that additional genetic or environmental factors contribute to the variability in severity observed in DYT-VPS16. Of note, reduced penetrance, which can be regarded as extremely late age at onset, has also been reported for DYT-VPS16.[19] Two of the twelve identified, presumably pathogenic *VPS16* variants were missense variants, which have only rarely been described.[15] The identification of a *de novo* missense variant, in addition to a previously reported one,[20] supports the role of missense variants in DYT-VPS16.

Findings in combined dystonia genes encompassed *ATP1A3*, *GCH1*, *GNAO1*, and *SGCE*, generally aligning with the phenotypic spectrum described in the literature for each gene. Unusual presentations included a DYT/PARK-GCH1 patient with macrocephaly in addition to dopa-responsive dystonia. However, the possibility that the unusual additional phenotype is caused by another genetic variant (dual molecular diagnosis) cannot currently be excluded. Among the 18 DYT/MYC-SGCE patients, 13 exhibited typical myoclonus-dystonia phenotypes, whereas in the remaining five, myoclonus was either absent or not clearly documented. *SGCE* variants can also manifest as isolated dystonia, albeit rarely.[14] *GNAO1* variants are typically linked to severe combined chorea-dystonia, but recent studies have identified splice site variants and haploinsufficiency linked to milder phenotypes and isolated dystonia.[21,22] Interestingly, in our study, a patient with a novel splice site variant presented solely with adolescence-onset cervical dystonia, consistent with these recent observations.

Variants in *ATP1A3* show a broad phenotypic spectrum, complicating genotype-phenotype evaluations. Two of our five patients experienced a rapid onset of dystonic symptoms in adolescence, a known presentation for *ATP1A3*, although typically accompanied by parkinsonism.[23] One of these patients also had deafness, often associated with *ATP1A3* in the context of CAPOS syndrome (Cerebellar ataxia, areflexia, pes cavus, optic atrophy, and sensorineural hearing loss).[24] Notably, this patient carried the same variant (p.Glu818Lys) previously described in CAPOS. Four of the five patients had isolated dystonia. This includes a patient with hemidystonia and the known pathogenic variant p.Thr613Met, which has only been associated with rapid-onset dystonia-parkinsonism until now.[25] These findings suggest that isolated dystonia can be part of the broad phenotypic spectrum of *ATP1A3*. Interestingly, we identified a heterozygous nonsense variant in *ATP1A3* in a patient with adult-onset, slowly progressive cervical dystonia and tremor – remarkable given that only one of 168 previously reported *ATP1A3* variants was truncating, with all others being missense.[23] The pathogenicity of heterozygous nonsense variants is supported by 1) the observation that heterozygous knockout mice develop dystonia,[26] (2) the gene's intolerance to LOF (pLI=1), and (3) the fact that the mechanism behind disease-associated missense variants has been shown to involve LOF.[27]

Findings in genes linked to dystonia with additional neurological or systemic features included *ACTB*, *IRF2BPL*, *SPR*, and *TUBB4A*, with phenotypes generally aligning with previous reports. One case involved generalized dystonia and deafness due to a known *ACTB* variant (p.Arg183Trp), while another patient with a *de novo* frameshift variant in *IRF2BPL* (p.Gln167*) exhibited generalized dystonia, learning disability, spasticity, and gait dysfunction. Additionally, a known homozygous missense variant in *SPR* (p.Arg150Gly) was linked to

infancy-onset, dopa-responsive upper limb dystonia, along with depression and an anxiety-related disorder. Although *SPR* is typically associated with dystonia, parkinsonism, and developmental delay, isolated dystonia has been observed in 3% of cases,[28] predominantly affecting the upper limbs, consistent with our patient's presentation. *TUBB4A* variants are associated with a broad phenotypic spectrum, including isolated dystonia, typically laryngeal, cervical, or upper limb dystonia,[29] as seen in three patients in this study.

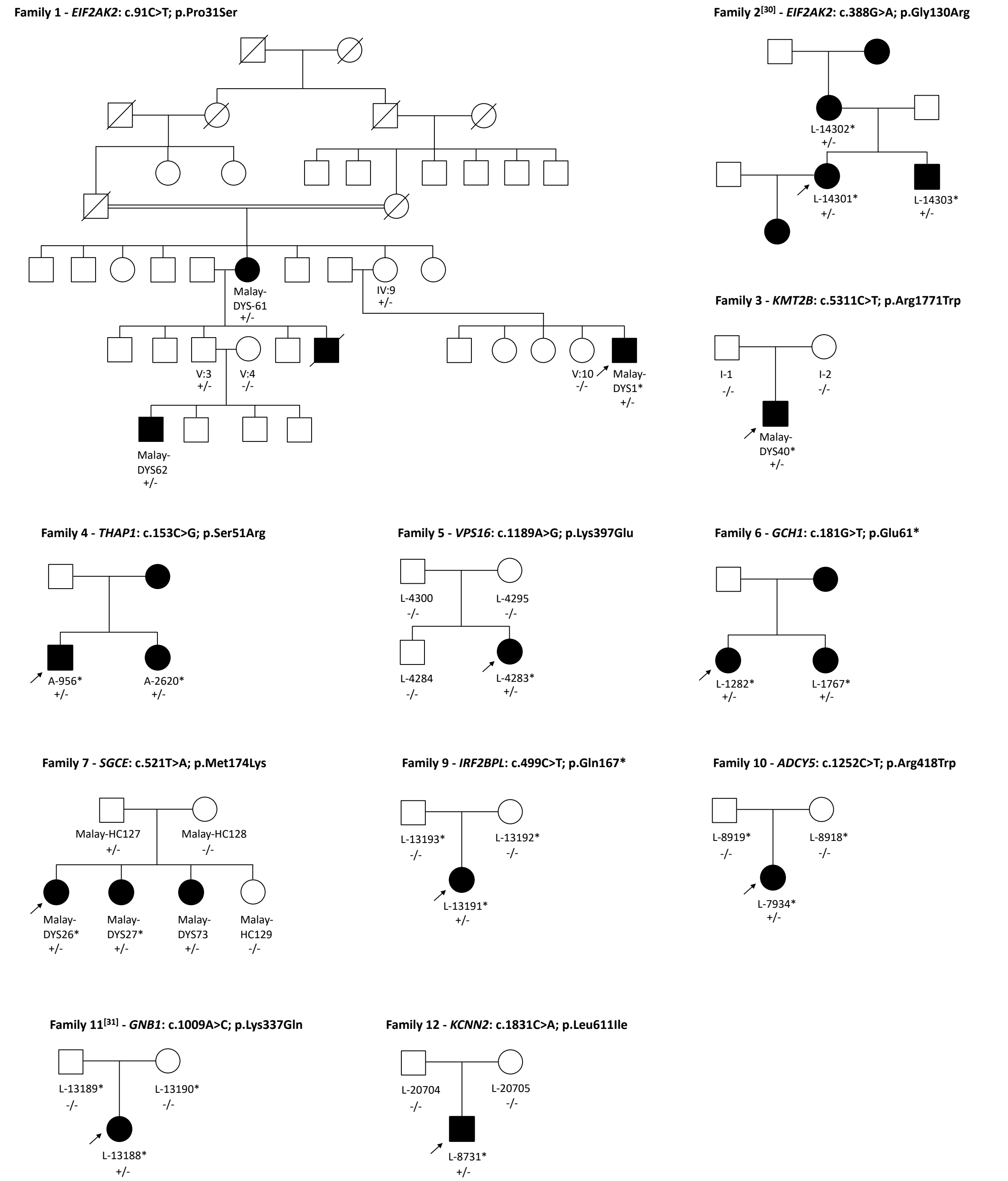

Supplementary Figure S1. Pedigrees of families in which exome sequencing identified a disease-causing variant, and DNA from family members was available for segregation analysis. Squares represent males, and circles represent females. Filled symbols indicate individuals with dystonia, and individuals with a diagonal line through their symbol are deceased. Index patients are marked with arrows. Mutation status is indicated as follows: "+" denotes the pathogenic variant, while "–" denotes the wildtype allele. Individuals marked with an asterisk underwent exome sequencing, whereas those with an ID without an asterisk were tested for the respective variant only by Sanger sequencing. Family 2 and 11 were previously published, with reference provided.

Supplementary References

1. Alganmi N, Abusamra H. Evaluation of an optimized germline exomes pipeline using BWA-MEM2 and Dragen-GATK tools. *PLoS One*. 2023;18(8):e0288371. doi:10.1371/journal.pone.0288371
2. McKenna A, Hanna M, Banks E, et al. The Genome Analysis Toolkit: a MapReduce framework for analyzing next-generation DNA sequencing data. *Genome Res*. 2010;20(9):1297-1303. doi:10.1101/gr.107524.110
3. Poplin R, Chang PC, Alexander D, et al. A universal SNP and small-indel variant caller using deep neural networks. *Nat Biotechnol*. 2018;36(10):983-987. doi:10.1038/nbt.4235
4. Kircher M, Witten DM, Jain P, O'Roak BJ, Cooper GM, Shendure J. A general framework for estimating the relative pathogenicity of human genetic variants. *Nat Genet*. 2014;46(3):310-315. doi:10.1038/ng.2892
5. Kj K, Lc F, G T, et al. The mutational constraint spectrum quantified from variation in 141,456 humans. *Nature*. 2020;581(7809). doi:10.1038/s41586-020-2308-7
6. Medvedev A, Lebedev M, Ponomarev A, et al. GRAPE: genomic relatedness detection pipeline. *F1000Res*. 2022;11:589. doi:10.12688/f1000research.111658.2
7. Ts K, I M. NgsRelate: a software tool for estimating pairwise relatedness from next-generation sequencing data. *Bioinformatics (Oxford, England)*. 2015;31(24). doi:10.1093/bioinformatics/btv509
8. Seidman DN, Shenoy SA, Kim M, et al. Rapid, Phase-free Detection of Long Identity-by-Descent Segments Enables Effective Relationship Classification. *Am J Hum Genet*. 2020;106(4):453-466. doi:10.1016/j.ajhg.2020.02.012
9. Huff CD, Witherspoon DJ, Simonson TS, et al. Maximum-likelihood estimation of recent shared ancestry (ERSA). *Genome Res*. 2011;21(5):768-774. doi:10.1101/gr.115972.110
10. Korneliussen TS, Albrechtsen A, Nielsen R. ANGSD: Analysis of Next Generation Sequencing Data. *BMC Bioinformatics*. 2014;15(1):356. doi:10.1186/s12859-014-0356-4
11. Manichaikul A, Mychaleckyj JC, Rich SS, Daly K, Sale M, Chen WM. Robust relationship inference in genome-wide association studies. *Bioinformatics*. 2010;26(22):2867-2873. doi:10.1093/bioinformatics/btq559
12. Mirza-Schreiber N, Zech M, Wilson R, et al. Blood DNA methylation provides an accurate biomarker of KMT2B-related dystonia and predicts onset. *Brain*. 2022;145(2):644-654. doi:10.1093/brain/awab360
13. Pidsley R, Zotenko E, Peters TJ, et al. Critical evaluation of the Illumina MethylationEPIC BeadChip microarray for whole-genome DNA methylation profiling. *Genome Biol*. 2016;17(1):208. doi:10.1186/s13059-016-1066-1
14. MDSGene. MDSGene. www.mdsgene.org/methods
15. Thomsen M, Lange LM, Klein C, Lohmann K. MDSGene: Extending the List of Isolated Dystonia Genes by VPS16, EIF2AK2, and AOPEP. *Mov Disord*. Published online January 20, 2023. doi:10.1002/mds.29327
16. Lange LM, Junker J, Loens S, et al. Genotype-Phenotype Relations for Isolated Dystonia Genes: MDSGene Systematic Review. *Mov Disord*. 2021;36(5):1086-1103. doi:10.1002/mds.28485
17. Park J, Reilaender A, Petry-Schmelzer JN, et al. Transcript-Specific Loss-of-Function Variants in VPS16 Are Enriched in Patients With Dystonia. *Neurol Genet*. 2022;8(1):e644. doi:10.1212/NXG.0000000000000644
18. Monfrini E, Avanzino L, Palermo G, et al. Dominant VPS16 Pathogenic Variants: Not Only Isolated Dystonia. *Mov Disord Clin Pract*. 2024;11(1):87-93. doi:10.1002/mdc3.13927
19. Li X yao, Wang L, Guo Y, Wan X hua. Mutations in the VPS16 Gene in 56 Early-Onset Dystonia Patients. *Movement Disorders*. 2021;36(3):780-781. doi:10.1002/mds.28540

20. Gu X, Lin J, Hou Y, Zhang L, Shang H. De Novo Missense Mutation of VPS16 in a Chinese Patient with Generalized Dystonia with Myoclonus. *Mov Disord Clin Pract*. 2022;9(4):551-552. doi:10.1002/mdc3.13392
21. Lasa-Aranzasti A, Larasati YA, da Silva Cardoso J, et al. Clinical and Molecular Profiling in GNAO1 Permits Phenotype-Genotype Correlation. *Mov Disord*. Published online June 16, 2024. doi:10.1002/mds.29881
22. Krenn M, Sommer R, Sycha T, Zech M. GNAO1 Haploinsufficiency Associated with a Mild Delayed-Onset Dystonia Phenotype. *Mov Disord*. 2022;37(12):2464-2466. doi:10.1002/mds.29258
23. Vezyroglou A, Akilapa R, Barwick K, et al. The Phenotypic Continuum of ATP1A3-Related Disorders. *Neurology*. 2022;99(14):e1511-e1526. doi:10.1212/WNL.0000000000200927
24. Muthaffar OY, Alqarni A, Shafei JA, Bahowarth SY, Alyazidi AS, Naseer MI. Childhood-related neural genotype–phenotype in ATP1A3 mutations: comprehensive analysis. *Genes Genom*. 2024;46(4):475-487. doi:10.1007/s13258-023-01481-8
25. Pittock SJ, Joyce C, O'Keane V, et al. Rapid-onset dystonia-parkinsonism: a clinical and genetic analysis of a new kindred. *Neurology*. 2000;55(7):991-995. doi:10.1212/wnl.55.7.991
26. Ikeda K, Satake S, Onaka T, et al. Enhanced inhibitory neurotransmission in the cerebellar cortex of Atp1a3-deficient heterozygous mice. *J Physiol*. 2013;591(13):3433-3449. doi:10.1113/jphysiol.2012.247817
27. Weigand KM, Messchaert M, Swarts HGP, Russel FGM, Koenderink JB. Alternating Hemiplegia of Childhood mutations have a differential effect on Na(+),K(+)-ATPase activity and ouabain binding. *Biochim Biophys Acta*. 2014;1842(7):1010-1016. doi:10.1016/j.bbadis.2014.03.002
28. Weissbach A, Pauly MG, Herzog R, et al. Relationship of Genotype, Phenotype, and Treatment in Dopa-Responsive Dystonia: MDSGene Review. *Movement Disorders*. 2022;37(2):237-252. doi:10.1002/mds.28874
29. Bally JF, Kern DS, Fearon C, et al. DYT-TUBB4A (DYT4 Dystonia): Clinical Anthology of 11 Cases and Systematized Review. *Mov Disord Clin Pract*. 2022;9(5):659-675. doi:10.1002/mdc3.13452
30. Musacchio T, Zech M, Reich MM, Winkelmann J, Volkmann J. A Recurrent EIF2AK2 Missense Variant Causes Autosomal-Dominant Isolated Dystonia. *Ann Neurol*. 2021;89(6):1257-1258. doi:10.1002/ana.26081
31. Reyes NGD, Di Luca DG, McNiven V, Lang AE. Dystonia with myoclonus and vertical supranuclear gaze palsy associated with a rare *GNB1* variant. *Parkinsonism & Related Disorders*. 2023;106:105239. doi:10.1016/j.parkreldis.2022.105239
